# One Bullet Causing Five Holes, Laparoscopic Exploration with Repair: A Case Report and Review of the Literature

**DOI:** 10.1155/2020/8861270

**Published:** 2020-08-01

**Authors:** Hunter Jones, Hassan Ahmed

**Affiliations:** Texas Tech University Health Sciences Center Amarillo, 1400 S Coulter St., Amarillo, TX 79106, USA

## Abstract

**Introduction:**

The proper treatment of penetrating abdominal wounds has been a controversial topic, and the preferred regimen has evolved over time. In recent years, many trauma centers have started using diagnostic laparoscopy in stable trauma patients in an effort to reduce the incidence of nontherapeutic laparotomy. This is more commonly seen in solid organ injuries, and its role is less clearly defined for hollow visceral injuries. *Case Presentation*. A 19-year-old male presented with a gunshot wound (GSW) to the abdomen with mild peritoneal signs and computed tomography (CT) findings. Diagnostic laparoscopy was performed with the repair of five lacerations to intra-abdominal organs including the sigmoid colon, rectum, bladder, and small bowel. *Discussion*. To our knowledge, this is the first case report in the literature detailing such a GSW repair. Abdominal GSWs have been repaired laparoscopically in the past, but none have elaborated on the repair of multiple defects of bowel and/or bladder.

**Conclusion:**

Therapeutic laparoscopy can be considered in selected cases of penetrating abdominal trauma. Laparoscopy offers several advantages over laparotomy including decreased mortality, complication rate, and length of stay.

## 1. Introduction

The proper treatment of penetrating abdominal wounds, specifically gunshot wounds (GSWs), has been a controversial topic, and the preferred regimen has evolved over time. An exploratory laparotomy has long been the standard of care for these patients. This approach was based on the assumption of a high incidence of peritoneal violation and significant visceral damage (98%) [1]. In the past couple of decades, selective nonoperative management has gained popularity and has been shown to provide benefit in patients without signs of internal organ damage or deflating hemodynamic stability [2]. However, there is no argument that when these instances occur, some degree of surgical intervention is required. In recent years, many trauma centers have started using diagnostic laparoscopy in stable trauma patients in an effort to reduce the incidence of nontherapeutic laparotomy [3]. The practice of laparoscopy in trauma has also started to play a more important role aside from screening and diagnostics, now being used as a therapeutic tool. This is more commonly seen in solid organ injuries, and its role is less clearly defined for hollow visceral injuries [4].

## 2. Case Presentation

**Figure 1:**
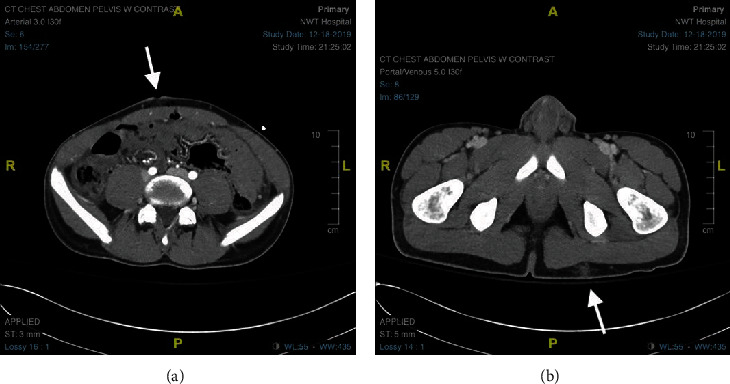
(a) CT scan of the abdomen/pelvis with bullet entrance point. (b) CT scan of the abdomen/pelvis with bullet exit point.

A 19-year-old male presented to the emergency room as a level 1 trauma with a self-inflicted GSW to the abdomen. The patient was attempting to put his 380 caliber handgun on safety when it discharged. On physical exam, the patient's abdomen was rigid and tender with a presumed entry point in the periumbilical area (Figure 1(a)) and a presumed exit point in the left posterior superior buttock (Figure 1(b)). No other injuries were present. The patient's measurements included a height of 165 cm and a weight of 60.1 kg (BMI 22.1). The vital signs upon arrival were BP 132/66 mmHg, pulse 106 bpm, and GCS 15/15. The patient's blood pressure decreased slightly to 101/45 mmHg; however, this corrected with intravenous (IV) fluids.

Computed tomography (CT) was performed and showed a mild amount of free fluid and air in the abdomen and pelvis (Figure 1(a)). There was no evidence of great vessel injury. Given the known trajectory of the bullet and lack of severity in the CT findings, diagnostic laparoscopy was pursued with readiness to convert to laparotomy if necessary.

**Figure 2:**
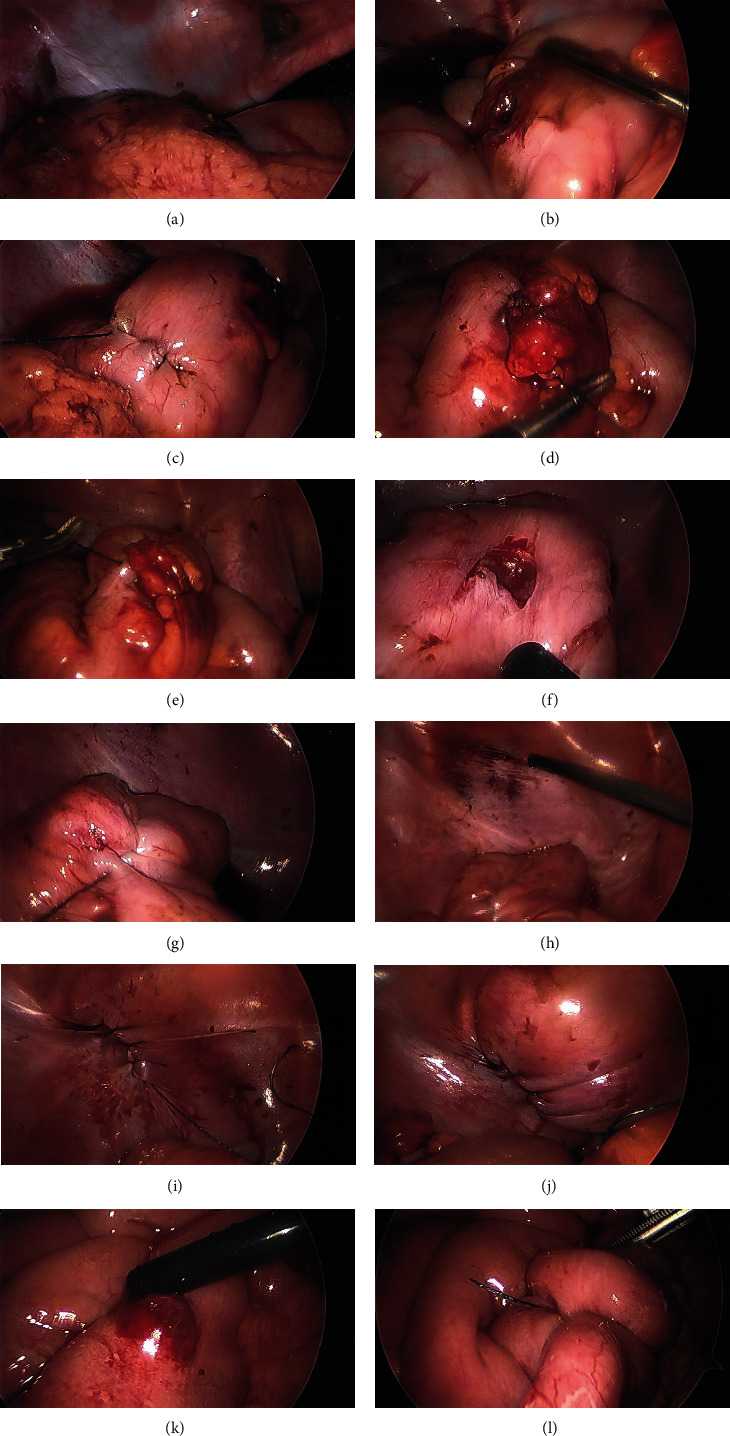
(a) Intraoperative laparoscopic visualization of stool contamination adjacent to the sigmoid colon. (b) Intraoperative laparoscopic visualization of first sigmoid perforation. (c) Intraoperative laparoscopic visualization of the sigmoid colon after repair of first perforation; second perforation visualized on the right. (d) Intraoperative laparoscopic visualization of the sigmoid colon during repair of second perforation. (e) Intraoperative laparoscopic visualization of the sigmoid colon following repair of second perforation. (f) Intraoperative laparoscopic visualization of rectal wall laceration. (g) Intraoperative laparoscopic visualization of the rectal wall following repair of laceration. (h) Intraoperative laparoscopic visualization of bladder laceration. (i) Intraoperative laparoscopic visualization of the bladder following repair of laceration. (j) Intraoperative laparoscopic visualization of the bladder with distillation for assessment of leak. (k) Intraoperative laparoscopic visualization of midsmall bowel perforation. (l) Intraoperative laparoscopic visualization of midsmall bowel following repair of perforation.

An initial periumbilical incision was made, and the abdomen was entered through a 5 mm port utilizing the Optiview technique. Pneumoperitoneum was obtained to 15 mmHg. Upon laparoscopic entrance, a small amount of blood and stool was noted in the left lower quadrant (Figure 2(a)). Additional ports were placed in the left lower quadrant (5 mm port) and right upper quadrant (11 mm port), both using the same optical trocar insertion method. Initial inspection of the sigmoid colon revealed a perforation with minimal contamination from stool spillage (Figure 2(b)). This was cleared and washed out followed by intracorporeal repair using 2-0 V-Loc™ suture in a running fashion done in 2 layers (Figure 2(c)). A second sigmoid perforation was found in the 4-5 cm distal to the first perforation as was repaired in a similar fashion (Figures 2(d) and 2(e)). The sigmoid was followed systematically to the pelvis, and a rectal wall laceration was noted (Figure 2(f)). It was found to penetrate the serosa and muscle layer but lack mucosal involvement. This was again repaired using 2-0 V-Loc™ suture in 2 layers (Figure 2(g)). After advancing down the rectum, a laceration was noted in the peritoneum over where the bladder was located (Figure 2(h)). Of note, preoperative urinary catheterization yielded hematuria and this laparoscopic finding confirmed bladder injury. The on-call urologist was contacted intraoperatively, and it was agreed upon for the primary surgeon to repair it in 2 layers with 2-0 V-Loc™ sutures and leave a Foley in place for 2 weeks postoperatively (Figures 2(i) and 2(j)). The colon was then systematically examined proximally including the rest of the sigmoid, descending colon, transverse colon, ascending colon, and cecum with no additional injuries noted. The ileocecal valve was identified; the small bowel was then run from the terminal ileum proximally. Another perforation was found approximately midsmall bowel with an enterotomy measuring less than 1 cm in size (Figure 2(k)). This was closed in 2 layers using 2-0 V-Loc™ suture (Figure 2(l)). The rest of the small bowel was run up to the ligament of Treitz with no additional injuries found. Of note, all the mesentery was examined while running the bowels with no significant injuries noted. All solid organs were intact on inspection. After abdominal and pelvic washout was performed, a size 19 French Blake drain was placed in the pelvis exiting through the left lower quadrant port site.

The GSW to the abdominal wall itself was examined. The bullet trajectory was angled in such a way that when it penetrated the skin, there was about a 5 cm difference from the presumed skin entry point and peritoneum entry point. Given this tangential path, the wound was not closed due to a low risk of herniation. The tract was thoroughly irrigated. Port sites were closed and dressings were placed. The patient tolerated the procedure well and was transferred to the postanesthesia care unit following extubation.

Postoperatively, the patient was placed in the surgical intensive care unit. There he progressed well and remained NPO with a NG tube in place. A urologist recommended the Foley catheter remains in place for 7 to 10 days with a cystogram done prior to removal. The patient was started on ertapenem upon his arrival and remained on IV antibiotics throughout his admission. Repeat CT with oral, rectal, and IV contrast on POD 5 was negative for any occult injury or contrast leak. Following this, the patient was started on a clear liquid diet with progression to full liquids. The patient was tolerating this well and was having normal bowel movements. He was advanced to a soft diet the following day. The patient's drain remained serosanguineous in nature and was able to be removed. The patient was discharged home on POD 6 with a Foley catheter in place, which was removed by a urologist 5 days later.

## 3. Discussion

Exploratory laparotomy has traditionally been the standard of care for diagnostic evaluation and treatment of patients with penetrating abdominal trauma. Despite the high versatility and accuracy for diagnosing and treating these types of injuries, some patients have no abdominal injuries present resulting in a nontherapeutic laparotomy (NL). These are associated with unnecessary complications in up to 41% of patients [5, 6]. In a more recent large data study (*n* = 4,520), Shamim et al. found that when compared to diagnostic laparoscopy (DL), NL was associated with increased mortality (OR 4.5), a higher rate of complications (OR 2.2), and a longer hospital stay (OR 2.7). NL was also associated with higher rates of pneumonia, venous thromboembolism (VTE), acute respiratory distress syndrome (ARDS), and myocardial infarction (MI) [3]. There is a similar contrast when comparing therapeutic laparotomy to therapeutic laparoscopy in patients with a positive abdominal injury. In a study of 518 patients, Chestovich et al. showed that length of stay was shorter in the therapeutic laparoscopy group than that in the therapeutic laparotomy group (4 days vs. 2 days). Wound infections were more common with open exploration (10.4% vs. 0%) as was the development of ileus or small bowel obstruction (9.4% vs. 1.1%) [7].

For laparoscopy to be useful in treating traumatic injuries, it must be safe, efficient, and reliable for diagnostic purposes as well as provide therapeutic value in selected patients. Although several reports have described laparoscopic exploration and treatment for traumatic injuries, there is still hesitancy among the trauma community to embrace it. This likely stems from multiple factors, including early reports of missed injuries, perceived inability to visualize all areas of the abdomen, and increased operative time, which are of special concern during periods of high trauma volume [8, 9]. Advanced training in minimally invasive surgery is becoming more common, however, and can help to mitigate the burden that these factors have on trauma specialists.

To our knowledge, this is the first case report in the literature detailing such a GSW repair. Abdominal GSWs have been repaired laparoscopically in the past, specifically with relation to the adrenal gland [4] and abdominal wall [10], but none have elaborated on the repair of multiple defects of bowel and/or bladder. While the role of laparoscopy is expanding, its role for visceral hollow organ repair has been less well defined. This is likely due to the fact that laparoscopy has been shown to have a decreased sensitivity with identifying hollow organ defects [11]. In cases of detected small bowel injury and insufficient laparoscopic experience, a laparoscopically assisted procedure can be chosen to avoid a complete laparotomy [12]. A systematic intraoperative approach and proper training in minimally invasive surgery can extenuate the possibility of missing hollow organ defects as well as decrease the likelihood of conversion into an open laparotomy.

## 4. Conclusion

Although the data are still controversial, the importance of laparoscopic technique is increasing in cases of penetrating trauma to the abdomen. This is true even with cases of extensive visceral hollow organ damage. Laparoscopy offers several advantages over laparotomy including decreased mortality, complication rate, and length of stay. However, laparoscopy should only be performed by experienced surgeons on properly selected patients.

## Data Availability

No data were used to support this study.
